# A comprehensive meta-analysis on the association between vitamin C intake and gestational diabetes mellitus: Insights and novel perspectives

**DOI:** 10.1097/MD.0000000000034740

**Published:** 2023-08-11

**Authors:** Lili Zhou, Junbo Liu, Min Zhou

**Affiliations:** a Department of Gynecology, The First Affiliated Hospital to Changchun University of Chinese Medicine, Changchun, Jilin Province, China.

**Keywords:** dietary intake, gestational diabetes mellitus, meta-analysis, pregnancy, vitamin C

## Abstract

Gestational Diabetes Mellitus (GDM) is a significant health concern in pregnant women and their offspring. Although Vitamin C is known to play a role in maintaining normal physiological processes, its relationship with GDM has not been fully elucidated.

**Methods:**

We conducted a systematic review and meta-analysis following the Preferred Reporting Items for Systematic Reviews and Meta-Analyses guidelines, analyzing data from 15 studies selected from PubMed, Embase, Web of Science, and the Cochrane Library up to May 16, 2023. These studies were selected based on inclusion criteria such as study design, outcome of interest, exposure factor, and data extractability. Quality assessment was performed using the Newcastle-Ottawa Scale. We assessed the heterogeneity between studies and conducted a sensitivity analysis.

**Results:**

Data from 10,131 subjects, including 1304 diagnosed GDM cases, were analyzed. The meta-analysis showed that women in the low Vitamin C exposure group had higher odds of developing GDM (odds ratio 2.72, 95% CI:1.24–4.19). There was a greater likelihood of increased GDM risk with lower Vitamin C exposure (standardized mean difference: −0.71, 95% CI [−1.07 −0.36]). Subgroup analysis revealed that both internal and external Vitamin C exposure, along with exposure during the second or third trimester of pregnancy, was associated with higher GDM incidence rates under low Vitamin C exposure. Sensitivity analysis confirmed the robustness of the results, and no significant publication bias was detected.

**Conclusion:**

Low Vitamin C exposure during pregnancy may increase the risk of GDM. Given these findings, it could be beneficial for pregnant women to increase their intake of Vitamin C-rich foods and to ensure adequate blood Vitamin C levels as a preventive measure against GDM.

## 1. Introduction

Gestational diabetes mellitus (GDM), characterized by an aberration in glucose homeostasis first recognized during pregnancy, is a burgeoning health issue with a global footprint,^[1,2]^ and it is a condition poised in the complex intersection of maternal metabolic physiology and pregnancy-induced adaptations, threatening both maternal and fetal health outcomes. Mother complications range from gestational hypertension and elevated Caesarean section rates to long-term susceptibility to type 2 diabetes. For fetuses and neonates, associated risks include congenital anomalies, macrosomia, neonatal hyperbilirubinemia, and an increased lifetime risk of obesity, type 2 diabetes, and metabolic syndrome.^[3]^ The myriad of health challenges posed by GDM underscores the urgency for a robust investigation of potential preventive strategies.

In 2019, the International Diabetes Federation reported that the global prevalence of GDM was 12.8%.^[4]^ Interestingly, the prevalence of GDM in China surpassed the global average, with an alarming rate of 14.8%.^[5]^ Despite its broad prevalence, the precise etiology of GDM remains under debate, with various interplaying factors such as genetic predisposition, lifestyle patterns, and environmental exposures. Emerging evidence highlights the role of oxidative stress, which is capable of interfering with insulin secretion and glucose metabolism, as a significant player in the pathogenesis of GDM.^[6,7]^ Studies have shown a heightened presence of oxidative stress biomarkers, such as markers of DNA damage and lipid peroxidation products, in the blood of women diagnosed with GDM.^[8,9]^ This evidence implicates the potential utility of antioxidants in mitigating the risk of GDM, thereby directing attention towards dietary sources of antioxidants as part of a GDM prevention strategy. Vitamin C (VC) is a dietary antioxidant. As a water-soluble vitamin, it plays diverse roles in human health, including anti-inflammatory activities, immune regulation, and involvement in various metabolic processes. The therapeutic potential of vitamin C in type 2 diabetes has been well-established in the literature, with studies confirming its beneficial effects on glucose metabolism and insulin sensitivity.^[10]^

However, the current body of research regarding the association between vitamin C intake and GDM risk is surprisingly sparse, and the available studies present inconsistent findings. Recognizing this gap in the literature, the present study aimed to conduct a comprehensive meta-analysis of observational studies evaluating the relationship between vitamin C exposure and the risk of GDM development. The objective was to unravel the potential association, if any, between these 2 entities. A clear understanding of this relationship can help formulate evidence-based nutritional recommendations for pregnant women, thereby providing a theoretical basis for GDM prevention. This research contributes significantly to the broader pursuit of maternal and neonatal health optimization by providing insights into the role of vitamin C in GDM.

## 2. Materials and methods

### 2.1. Search strategy

During the systematic review process and subsequent reporting of our results, we adhered to the Preferred Reporting Items for Systematic Reviews and Meta-Analyses guidelines.^[11]^ Two researchers conducted a systematic search for pertinent studies, independently determined their eligibility, extracted data, and evaluated the quality of the research. Four electronic databases, PubMed, Embase, Web of Science, and Cochrane Library, were searched on May 16, 2023, and no time limitation was applied. The vocabulary and syntax were adapted according to the database. PubMed search terms were as follows: (“Gestational Diabetes Mellitus” [Mesh] odds ratio [OR] “gestational diabetes mellitus” [tiab] OR “pregnancy-induced diabetes” [tiab]) AND (“Ascorbic Acid” [Mesh] OR “ascorbic acid” [tiab] OR “vitamin C” [tiab]). The reference lists of the relevant articles were also manually screened for any additional possible records.

### 2.2. Inclusion criteria

1. Population: Women of child-bearing age.
2. Exposure: Dietary intake of Vitamin C (not including Vitamin C supplements) or blood Vitamin C concentration.
3. Comparison: Comparisons between different Vitamin C exposure levels.
4. Outcome: Diagnosis of GDM.
5. Study Design: Case-control studies or cohort studies.

### 2.3. Exclusion criteria

1. Studies that had been published multiple times or those from which data could not be obtained.
2. Studies with poor quality.
3. Case reports, commentaries, expert opinion, and narrative reviews.
4. Cross-sectional studies.
5. Multi-factor studies such as those involving multivitamin intake and gestational diabetes.

### 2.4. Data extraction

The literature screening and data extraction shall be carried out independently by 2 evaluators and cross-checked; if there are differences, the differences will be discussed and resolved. The data to be extracted included the author(s) of the study, year of publication, location of the study, design type of the study, size of the study population, number of GDM cases, standards used for GDM diagnosis, type and detection method of exposure, number of individuals in the exposed and nonexposed groups, mean values, standard deviations, or quartiles of Vitamin C exposure in the GDM and non-GDM groups, adjusted or unadjusted OR or RR values, along with their corresponding 95% CI. When there was no data of interest in the published report, we contacted the investigators of the original study by email to request unpublished data.

### 2.5. Quality assessment

The quality of the studies included in our meta-analysis was rigorously evaluated by 2 independent reviewers using the Newcastle-Ottawa Scale (NOS).^[12]^ NOS is a well-established tool that rates studies based on 9 components spread across 3 crucial categories: selection, comparability, and outcome. These categories enabled us to assess the possible sources of bias inherent to the studies. Following this comprehensive evaluation, each study was assigned a quality score that ranged from 0 to 9. The interpretation of these scores is as follows: studies receiving a score from 0 to 3 are considered to be of low quality; those scoring between 4 and 6 are regarded as being of moderate quality; and those scoring from 7 to 9 are deemed high-quality research.

### 2.6. Statistical analyses

The effect measures used for this meta-analysis will be the OR and standardized mean difference (SMD), each with their respective 95% CI. Heterogeneity between the included studies was evaluated using the *I*^2^ and Q tests. If the *P* value exceeds .05, it indicates that the heterogeneity is not statistically significant, and thus, a fixed effects model will be adopted. Conversely, if the *P* value is less than or equal to .05, indicating significant heterogeneity, a random effects model will be employed. To explore the sources of heterogeneity, subgroup analyses were conducted based on the type of Vitamin C exposure, geographic location of the studies, period of VC exposure, and diagnostic criteria for GDM. Sensitivity analysis was performed using the 1 study-removed approach to assess the stability of the results. Finally, potential publication bias was assessed through visual inspection of funnel plots and Egger linear regression test. A 2-sided *P* < .05 was considered statistically significant in all statistical tests. Stata version 17 (StataCorp, College Station, TX) was used for the data analysis.

## 3. Results

### 3.1. Search results and study selection

**Figure 1.**
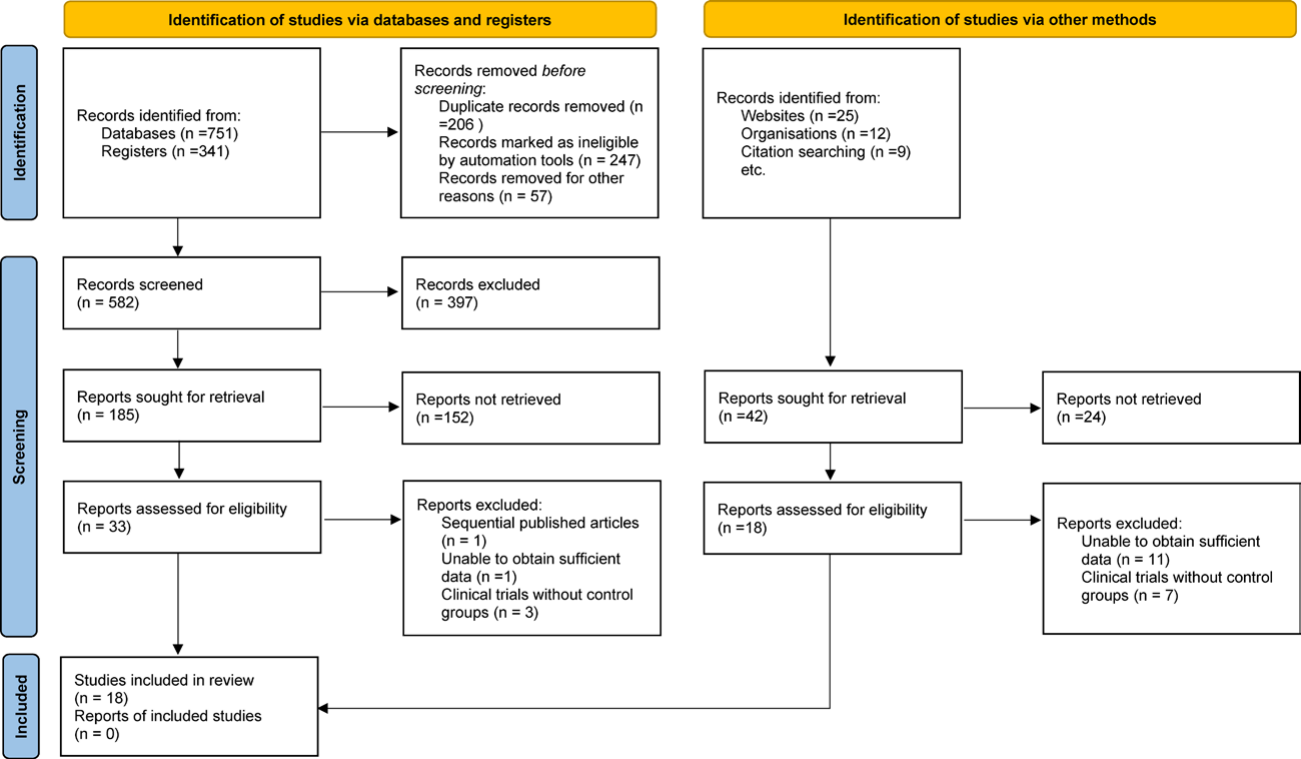
Selection process of included studies.

From the initial search of the electronic databases, 1092 related literatures were found. After removing repetitive literature, reading titles and abstracts, and screening strictly according to the inclusion and exclusion criteria, 33 related studies were obtained and 18 were excluded from further reading. Ultimately, 15 articles were included.^[13–27]^ The literature screening process and the results are shown in Figure 1.

### 3.2. Study characteristics

Fifteen articles from various countries, including India, China, and the United States, published between 2004 and 2020, were reviewed. The majority of these articles (13) were case-control studies, whereas the remaining 2 were cohort studies. The collective data of these studies encompassed 10,131 subjects, of which 1304 were diagnosed with GDM. It should be noted that the methods of GDM diagnosis varied across different studies, as did the types of Vitamin C exposure and their respective measurement techniques. Further details of these variations are listed in Table 1.

**Table 1:** Characteristics of studies included in the meta-analysis.

Study (yr)	Country	Study design	GDM diagnosis criteria	Sample size (GDM count)	VC exposure assessment	VC exposure period
Cuilin Zhang et al (2004)	USA	Cohort study	NDDG	755 (33)	Internal exposure (colorimetry)	Average 13 wk of pregnancy
Fabiane Rodrigue et al (2018)	Brazil	Case-control	ADA	78 (48)	Internal exposure (spectrophotometry)	Late pregnancy
Hedyeh Masoodi et al (2015)	India	Case-CONTROL	IADPSG	200 (42)	Internal exposure (spectrophotometry)	Mid-pregnancy
Min Shang et al (2015)	China	Case-control	IADPSG, ADA	68 (28), 68 (10)	Internal exposure (colorimetry)	Late pregnancy
Mohd Suhail et al (2010)	India	Case-control	ADA	46 (23)	Internal exposure (colorimetry)	At birth
Simmi Kharb (2008)	India	Case-control	WHO	50 (25)	Internal exposure (NA)	Late pregnancy
Surapaneni K M, Vishnu Priya V (2008)	India	Case-control	O’Sullivan standard (1964)	40 (20)	Internal exposure (Tietz method)	Late pregnancy
Chaoqun Liu et al (2020)	China	Prospective cohort study	IADPSG	3009 (344)	External exposure (FFQ)	Mid-pregnancy
Hee-Jin Park et al (2013)	South Korea	Case-control	Carpenter & Coustan test (1982)	263 (44)	External exposure (3-d food record)	Pre pregnancy and post-pregnancy
Vida Mohammad Parast et al (2017)	Iran	Case-control	ADA	80 (40)	External exposure (FFQ)	Pre pregnancy and post-pregnancy
Aleksandra Kozlowska et al (2018)	Poland	Case-control	Clinical history	57 (29)	External exposure (7d24h trained personnel interview)	Late pregnancy
Chauntelle Jack Roberts et al (2020)	USA	Case-control	Self-defined method	62 (33)	External exposure (7d 24 h trained personnel interview)	Mid and late pregnancy
Moniek Looman et al (2019)	Australia	Nested case-control	Self-reported	3607 (277)	External exposure (FFQ)	Pre pregnancy
Alanood Aljanahi et al (2020)	Saudi Arabia	Case-control	DCDM	121 (72)	External exposure (3-d food record)	Mid and late pregnancy
Simona Bo et al (2005)	Italy	Case-control	Carpenter & Coustan Test (1982)	420 (126)	External exposure (FFQ)	Mid and late pregnancy

ADA = American Diabetes Association, ACOG = American College of Obstetricians and Gynecologists, DCDM = diagnosis and classification of diabetes mellitus, GDM = gestational diabetes mellitus, IADPSG = International Association of Diabetes and Pregnancy Study Groups, NA = not available in the study, NDDG = National Diabetes Data Group criteria, VC = Vitamin C.

Self-defined Method: Blood sugar > 140 mg/dl in 50 g1 h and 100 g3 h glucose tolerance tests. Internal Exposure: Plasma/Red blood cell Vitamin C levels; External Exposure: Dietary intake of Vitamin C.

### 3.3. Results of quality assessment

We assessed the methodological quality of each Randomized Controlled Trial (RCT) using the NOS. In general, 1 study scored 7 points, 5 studies scored 8 points, and 9 studies scored 9 points. No studies were blinded and there was no evidence of allocation concealment. No funding bias was evident in any of the studies. No studies had incomplete outcome data, early stoppage bias, or baseline imbalances. The risks of bias and corresponding ratios are summarized (Table 2).

**Table 2:** The quality assessment according to NOS of each cohort study.

Study	Selection				Comparability	Outcome			Total score
	Representativ-eness of the exposed cohort	Selection of the nonexposed cohort	Ascertainment of exposure	Demonstration that outcome	Comparability of cohorts	Assessment of outcome	Was follow-up long enough	Adequacy of follow-up of cohorts	
Cuilin Zhang et al (2004)	★	★	★	★	★★	★	★	★	9
Fabiane Rodrigue et al (2018)		★	★	★	★★	★	★	★	8
Hedyeh Masoodi et al (2015)	★	★	★	★	★★	★	★	★	9
Min Shang et al (2015)	★	★	★	★	★★	★		★	8
Mohd Suhail et al (2010)	★	★	★	★	★★	★	★	★	9
Simmi Kharb (2008)	★	★	★	★	★	★	★	★	8
Surapaneni K M, Vishnu Priya V (2008)	★	★	★	★	★★	★	★	★	9
Chaoqun Liu et al (2020)	★	★	★	★	★★	★	★	★	9
Hee - jin Park et al (2013)	★	★	★	★	★★	★	★	★	9
Vida Mohammad Parast et al (2017)	★	★	★	★	★★	★	★	★	9
Aleksandra Kozlowska et al (2018)		★	★	★	★★	★	★	★	8
Chauntelle Jack Roberts et al (2020)	★	★	★	★	★★	★	★	★	9
Moniek Looman et al (2019)	★	★	★	★	★★	★		★	8
Alanood Aljanahi et al (2020)	★	★		★	★	★	★	★	7
Simona Bo et al (2005)	★	★	★	★	★★	★	★	★	9

NOS: Newcastle-Ottawa Scale.

### 3.4. The results of meta-analysis

**Figure 2.**
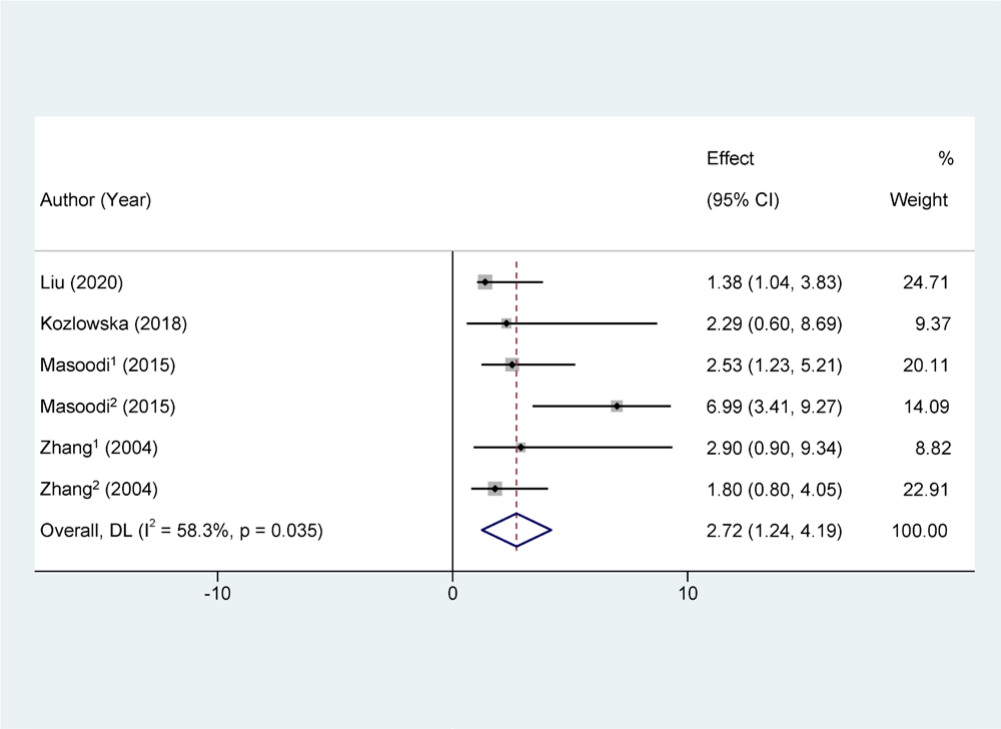
Forest plots of the relationship between vitamin C exposure levels and the risk of gestational diabetes mellitus.

The Results section of the analyzed 15 publications is as follows: Four publications demonstrated a relationship between various Vitamin C exposure levels and GDM, among which 2 simultaneously evaluated dietary and blood VC exposure levels.^[20,27]^ The results indicated that women in the low VC exposure group had a higher risk of developing GDM, with 3 results showing a statistical significance. The meta-analysis revealed statistically significant heterogeneity among the included studies (*I*^2^ = 58.3%, *P* = .035). After merging using a random effects model, the OR was 2.72 (95% CI:1.24–4.19), indicating that the incidence of GDM in the low VC exposure group was significantly higher than in the high exposure group. (Refer to Fig. 2).

**Figure 3.**
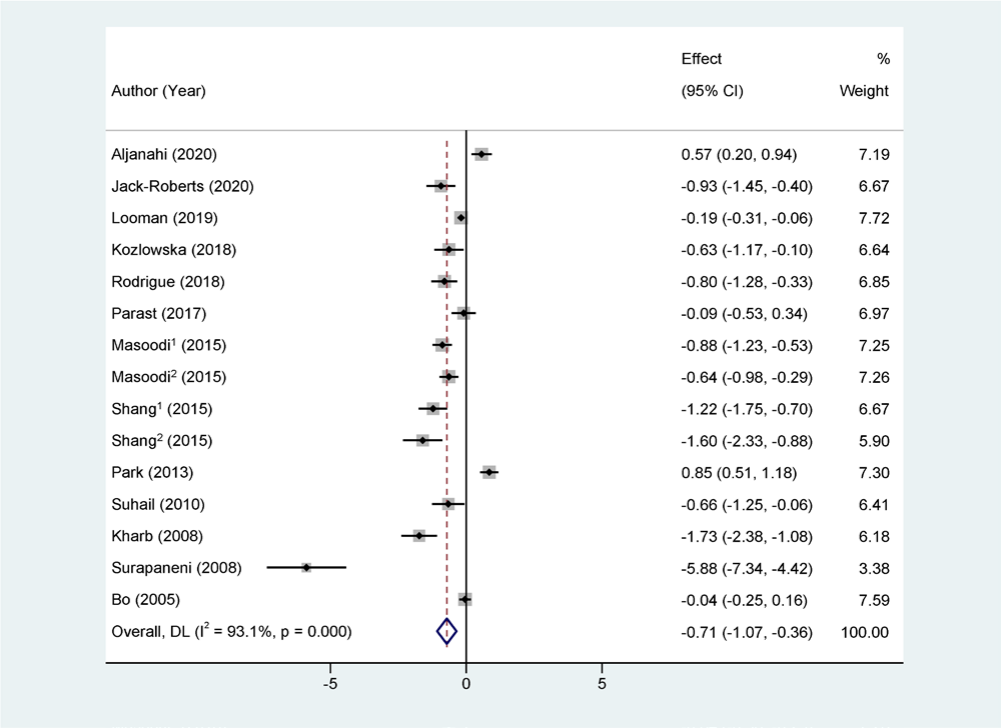
Forest plots of the association between the amount of vitamin C exposure and the risk of gestational diabetes mellitus.

Thirteen articles that included continuous VC exposure were merged. One^[24]^ of these studies employed 2 distinct diagnostic methods for GDM, and 1^[20]^ simultaneously assessed dietary and blood VC exposure levels. The meta-analysis revealed significant heterogeneity among these studies (*I*^2^ = 93.1%, *P* < .01). After applying the random effects model, the SMD was −0.71 (95% CI: −1.07 to −0.36), suggesting a greater likelihood of increased GDM risk with lower VC exposure. (Refer to Fig. 3).

### 3.5. The results of subgroup analysis

A subgroup analysis was conducted to study the relationship between the levels of VC exposure and GDM risk based on VC exposure type, VC exposure timing, and the research region. The OR results revealed that both internal and external VC exposure; regions in China, Europe, America, and India; and exposure during the second or third trimester of pregnancy were associated with higher GDM incidence rates under low VC exposure levels (Table 3).

**Table 3:** Subgroup analysis showing OR of GDM for low VC exposure.

Characteristic	Number of studies	Number of results	Heterogeneity test *I*^2^ (%)	*P* value	Model	OR (95% CI)
Exposure type						
Intra-exposure	2	2	0%	.87	Fixed effects	2.60 (1.45–4.95)
Extraexposure	4	4	80%	<.01	Random effects	2.58 (1.10–6.10)
Region						
China	1	1				1.40 (1.05–1.79)
Western Countries	2	3	0%	.83	Fixed effects	2.18 (1.20–3.95)
India	1	2	81%	.03	Random effects	4.85 (1.30–18.30)
Exposure period						
Pre pregnancy or early pregnancy	2	2	88%	.01	Random effects	4.20 (0.80–21.10)
Mid or late pregnancy	4	4	23%	.30	Fixed effects	1.65 (1.25–2.05)

GDM = gestational diabetes mellitus, OR = odds ratio, VC = Vitamin C.

Further subgroup analysis was performed considering the type of VC exposure, duration of exposure, research region, and GDM diagnostic criteria to examine the correlation between the amount of VC exposure and GDM risk. The SMD results indicated that internal exposure (blood VC concentration) in Europe, America, and India, and during the second or third trimester of pregnancy, lower VC exposure amounts were more likely to result in GDM under the American Diabetes Association (ADA) diagnostic criteria grouping. However, this relationship was not significant in other subgroups (Table 4).

**Table 4:** Subgroup analysis showing VC exposure concentration differences in GDM.

Characteristics	Number of studies	Number of outcomes	Heterogeneity Test (*I*^2^ %)	*P* value	Model	SMD (95% CI)
Exposure type						
Internal exposure	6	7	89	<.01	random effects	−1.73 (−2.88 to −0.50)
External exposure	10	12	83	<.01	Random effects	−0.06 (−0.34 to 0.27)
Region						
China	3	6	84	<.01		−0.37 (−1.00 to 0.30)
Europe and America	5	5	75	<.01	Random effects	−0.43 (−0.77 to −0.13)
India	4	5	91	<.01	Random effects	−1.86 (−3.66 to −0.05)
Exposure period						
Pre pregnancy or Early Pregnancy	6	6	90	<.01	Random effects	0.12 (−0.34 to 0.58)
Mid or late pregnancy	10	12	91	<.01	Random effects	−0.99 (−1.76 to −0.24)

GDM = gestational diabetes mellitus, SMD = standardized mean difference, VC = Vitamin C.

### 3.6. Sensitivity analysis

A sensitivity analysis was carried out by sequentially eliminating 1 study at a time and then recombining the remaining studies. The combined SMD ranged from −0.66 (95% CI: −1.16 to −0.18) to −0.36 (95% CI: −0.76 to 0.06), while the combined OR ranged from 1.68 (95% CI: 1.26–2.56) to 3.06 (95% CI:1.75–5.51). These results remained consistent across all iterations, implying that the results of the meta-analysis were stable and reliable.

### 3.7. Publication bias

**Figure 4.**
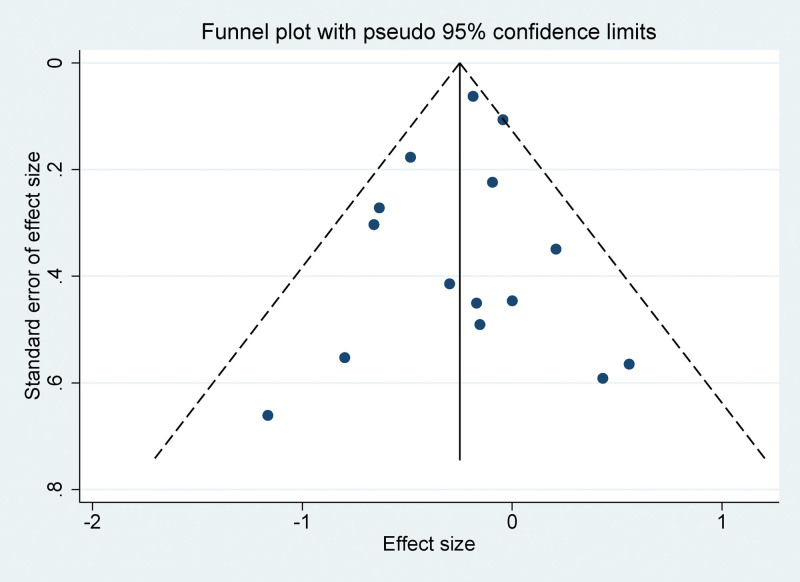
Funnel plot for publication bias in all included studies.

The funnel plots constructed in the observed study showed symmetry, and no significant publication bias was detected in the funnel plots (Fig. 4). Egger linear regression test indicated that no significant publication bias was detected in the meta-analyses under different variables (*P* > .05), thus further confirming the robustness of the meta-analysis results.

## 4. Discussion

Our study, for the first time, presents a meta-analysis of the association between Vitamin C exposure and GDM risk. Incorporating 15 observational studies, our findings indicate a significant risk of GDM development with low Vitamin C exposure. In the subgroup analysis, the OR results exhibited a heightened incidence of GDM with lower Vitamin C exposure across different demographics, regional clusters, and stages of pregnancy. Similarly, under the standards of the American Diabetes Association, the SMD results indicate that cases of low Vitamin C exposure have a higher prevalence of GDM during mid to late pregnancy.

Several potential mechanisms could elucidate how low exposure to Vitamin C increases the risk of GDM. First, animal studies have suggested that oral intake of Vitamin C can lower plasma glucose, insulin, and triglyceride levels in diet-induced obese rats.^[28]^ Second, Vitamin C may ameliorate insulin resistance induced by tumor necrosis factor-α in HepG2 cells by regulating the insulin receptor substrate /Protein Kinase B/glycogen synthase kinase 3β signaling pathway, thereby enhancing the expression of Glucose Transporter 2 protein. Third, a low concentration of Vitamin C may interfere with erythrocyte glucose transport, leading to hyperglycemia.^[29]^ Lastly, Vitamin C may enhance insulin sensitivity and reduce levels of endothelial adhesion molecules by mitigating oxidative stress.^[30]^

Our findings indicate potential variation in the relationship between Vitamin C concentration and GDM under different GDM diagnostic criteria. Regarding diagnostic criteria, the International Association of Diabetes and Pregnancy Study Groups standard only requires a single threshold to be exceeded in the Oral Glucose Tolerance Test during pregnancy, whereas the ADA standard requires exceeding 2 or more thresholds.^[24]^ Studies have shown that GDM diagnosis rate nearly doubled under the International Association of Diabetes and Pregnancy Study Groups standard.^[31]^ Therefore, it could be hypothesized that women diagnosed with GDM under the ADA standard might experience more severe oxidative stress, leading to a more noticeable decrease in Vitamin C concentration.^[24]^ Moreover, oxidative stress and antioxidant capacity in pregnant women vary from those in nonpregnant women and can differ across pregnancy stages.^[32]^ Hence, Vitamin C exposure during different pregnancy stages may affect oxidative stress in the body and, thus, the association with GDM.

Nonetheless, while evidence supports that increased Vitamin C levels can potentially reduce GDM risk, it is important to note that dietary Vitamin C and supplements are not substitutes for a comprehensive prenatal health plan. Regular physical activity, balanced diet, regular prenatal checkups, and monitoring of glucose levels remain crucial elements for managing pregnancy health and mitigating the risk of GDM. Furthermore, the context of nutritional epidemiology should be considered. Individual nutrient effects, such as Vitamin C, are complex and can be influenced by a plethora of factors.^[33]^ Nutrient-nutrient interactions, bioavailability, and even an individual’s genetic predisposition can affect the relationship between nutrient intake and disease outcome. Therefore, while the potential role of Vitamin C in mitigating GDM risk is promising, it should be viewed as part of a broader, more holistic approach to prenatal health. In addition, the potential of Vitamin C as a preventive factor for GDM should not overshadow the need for further research on GDM’s underlying mechanisms of GDM. Our understanding of GDM pathogenesis remains incomplete, and although we have indications that oxidative stress and inflammation may play a significant role, there are likely other contributing factors.^[34]^ Uncovering these issues can pave the way for more targeted and effective preventive strategies. The findings of this study underscore the importance of conducting further research, particularly RCTs, which are considered the gold standard in healthcare research. Vitamin C, as a dietary element, cannot be investigated in isolation; hence, future studies should also explore its interaction with other micronutrients and how these might collectively influence GDM risk.

Our study encompassed various Vitamin C exposure scenarios and conducted subgroup analyses for internal and external exposure. Internal exposure reflects overall multi-pathway exposure, potentially reducing the impact of individual differences on absorption and metabolism and providing a more accurate measure than external exposure. However, our study has some limitations. First, there was substantial heterogeneity between the included studies due to varying GDM diagnostic standards, Vitamin C exposure periods, and testing methods, which might influence the outcomes.^[2]^ Second, our study lacks RCTs focusing on Vitamin C and its relationship with GDM, and large-sample cohort studies are relatively scarce. Most of the included studies were case-control studies, which inherently have a lower quality of evidence.^[3]^ Third, in studies on external Vitamin C exposure, the role of Vitamin C supplements was generally overlooked, focusing only on dietary intake, which might have impacted the external exposure results.

## 5. Conclusion

In conclusion, low Vitamin C levels during pregnancy may increase the risk of GDM. Although further large-scale prospective cohort studies and RCTs are needed to confirm this relationship, based on the current evidence, it would be prudent to advise pregnant women to increase their intake of Vitamin C-rich foods and elevate blood Vitamin C concentration as a preventive measure against GDM, thereby promoting maternal and infant health.

## Author contributions

**Data curation:** Junbo Liu, Min Zhou.

**Formal analysis:** Junbo Liu.

**Methodology:** Lili Zhou.

**Resources:** Min Zhou.

**Software:** Lili Zhou.

**Writing – original draft:** Lili Zhou.
